# Collaboration: The Paradigm of Practice Approach between the Forensic Psychiatrist and the Forensic Psychologist

**DOI:** 10.3389/fpsyt.2012.00089

**Published:** 2012-11-15

**Authors:** Ernest Ayodele Gbadebo-Goyea, Hilary Akpudo, Cynthia D. Jackson, Tamer Wassef, Narviar C. Barker, Rhonda Cunningham-Burley, Shahid A. Ali, Shagufta Jabeen, Rahn Kennedy Bailey

**Affiliations:** ^1^Department of Psychiatry and Behavioral Sciences, Meharry Medical CollegeNashville, TN, USA

**Keywords:** forensic psychiatrist, forensic psychologist, competency to stand trial, sexually violent predator, mental state, child custody, criminal responsibility and trier of facts

## Abstract

The importance and relevance of forensic practice to societal evolution has increased exponentially in recent years. As society evolves in its understanding of the complex relationships between mankind and society, we rely more and more on the services of forensic experts. This article elucidates the professions of forensic psychiatry and forensic psychology. We examine the two distinct professions from the spectrum of collaboration, integration of services, differences, and similarities. We also compare and contrast the educational background and training requirements for these two professions; and present illustrative scenarios and real life examples of the daily functions of both professionals. Lastly, we present demographic data for the areas of employment, numbers, and geographic distribution of the two professions. Forensic psychiatry is the interface between medicine and law, while forensic psychology is the interface between psychology and law. As such, these professions are mired with complexities and challenged by vulnerabilities. Professionals from both fields can serve as expert witnesses in court and therefore face similar challenges in their course of professional practice. Collaboration between these two professions has the potential to increase both the credibility and utility of forensic services to the courts, the individuals served, and the general public.

## Introduction

Forensics refers to the application of science in the context of law (Chaplow et al., 1992)^1^. Forensic Psychiatry is a subspecialty of general psychiatry that focuses on the interface between psychiatry and the law as when psychiatric questions arise in a legal context or when legal questions arise in a psychiatric context (Pinals, 2005). This subspecialty was introduced in 1967 (Freedman, 1967; Morozov, 1967). Forensic psychiatry interfaces with criminology, penology, commitment of the mentally ill, compensation cases, the problems of releasing information to the court, and the issue of expert testimony. A forensic psychiatrist completes additional professional training that blends medicine and law. By contrast, forensic psychology is that branch of psychology that investigates the psychology of crime with particular reference to personality factors presented by the criminal (Grisso, 1987). Forensic psychologists analyze the criminal mind and intent, and offer treatment to the defendants as well as consultation to attorneys who engage in legal proceedings. There are similarities and differences between these two forensic professionals. They both can serve as expert witnesses in courts and in relation to medical-legal issues, though their educational background, service methods, and therapeutic approaches differ. Both of these forensic professions face the same challenges that threaten their professional credibility. Some of the ethical issues that face both professions as an expert witness are: matters of competence of the defendant, informed consent, confidentiality, multiple relationships, and special issues related to billing (Knapp and Vandercreek, 2001). Another role encompassed by the forensic psychologist is that of neuropsychologist. They work in clinical and forensic settings. Lawyers are increasingly seeking neuropsychological consultation on an expanding set of legal issues. Clinical neuropsychologists apply a scientific approach that meets judicial standards for expert testimony and assist the Trier of fact in judicial decisions. A Trier of fact is the individual(s) who make findings of fact – a jury or a judge in a bench trial (Binder and McNiel, 2007; Grover, 2010). This rapid growth in forensic consulting leads to the unrestrained discovery of raw data and psychological test materials during a client’s litigation. Over exposure to discovery data has the potential to erode its reliability and validity because of multitudinous examination and diverse interpretations (Kaufmann, 2009). For example, how much examination and interpretation must take place with the Trayvon Martin case before public opinion, bias, and conjecture erode the reliability and validity of actual findings? Collaborations between forensic psychiatrists and forensic psychologists can reduce potential compromise to the outcome of this case and maintain credibility in decision-making and discovery.

### Integrative services by forensic psychiatrists and forensic psychologists

The integrative approach to psychiatry has become the norm in recent years. Forensic psychiatry plays a very important role in contemporary integrative psychiatry. Integrative psychiatry uses both conventional and complementary therapies in the treatment of psychiatric disorders (Smalc et al., 2008; Lake et al., 2012). The integrative approach now is viewed as better and more ethical in forensic applications.

## Differences between the Forensic Psychiatrist and Forensic Psychologist

Forensic psychiatrists may prescribe medication, monitor lab results, order, and interpret brain scan and imaging studies and perform medical procedures. Forensic psychologists focus on non-pharmacological approaches such as cognitive behavioral therapy, eye movement desensitization and reprocessing (EMDR), systemic therapy, and stress inoculation therapy, to name a few. As a general rule, psychologists do not provide medications and are not licensed to write prescriptions. There are a limited number of psychologists who are authorized by the Department of Defense to provide prescription medications for the treatment of mental illness. Psychologists licensed in the states of New Mexico and Louisiana can provide such prescription medications but under a physician’s supervision. The statute in New Mexico allows licensed doctors of psychology who have passed additional training to prescribe medication. Four other states – Illinois, Georgia, Hawaii, and Tennessee – are considering similar legislation^2^.

Noted distinctions between psychiatrists and psychologists are their educational background and training. Psychiatry is a medical specialty that focuses on the study, diagnosis, and treatment of mental disorders (Rappeport, 1982). Forensic Psychiatry is a subspecialty of psychiatry and deals with the application of clinical psychiatry in the context of law. Forensic Psychiatrists apply their clinical skills for all professional work involving forensics (Appelbaum, 2010). The primary educational qualification to becoming a forensic psychiatrist is the M.D. degree, followed by residency training in general psychiatry and a fellowship in forensic Psychiatry. By contrast, psychology is an academic and applied discipline involved in the systematic study of mental functions and human behavior. Clinical Psychology is the application of this discipline for the purpose of understanding, preventing, and relieving subjective distress or dysfunction in humans, and is inclusive of the biopsychosocial environment. Forensic Psychology is the intersection between psychology and the law. Forensic Psychologists are professionals in their own right, and may have special expertise in topics not usually studied in detail by psychiatrists. These topics include psychological testing, a systemic approach to human behavior, and or genomic imprinting and inheritance traits. The educational qualifications for forensic psychologists are doctoral level degrees such as Ph.D., Psy.D, or Ed.D.

Forensic Psychologists have Ph.Ds in clinical or counseling psychology or the equivalent and have more training in psychological research and personality assessment than MDs. Forensic Psychiatrists are physicians or MDs who have completed at least four years of post graduate training plus an additional year of fellowship training (Table 1)^3^. They are the only mental health specialists licensed to prescribe medications and to give full physical examinations. The medical training of psychiatrists qualifies them for administering somatic therapies such electro-convulsive therapy and psychotropic medication. Forensic Psychologists work in some 40 different specialties. They may be researchers studying animal behavior or electrical impulses in nerve cells or environmental psychologists observing people in crowded cities. Forensic Psychiatrists also treat a variety of patients from children and adolescents with behavior disorders to adults who have mental illness. Forensic psychologists treat patients who have emotional and mental disorders with behavioral intervention. They conduct psychological testing, hypnosis, and counseling, which are critical in assessing a person’s mental state and in determining the most effective course of action^4^

**Table 1:** **Educational differences between psychiatrists and psychologists**.

Description	Forensic psychiatrist	Forensic psychologist
Degree	MD or DO	Masters
Advanced training	4 Year residency +1 year fellowship	Ph.D., Psy.D, or Ed.D

## Similarities between the Forensic Psychiatrist and Forensic Psychologist

In spite of the above differences in these professions, there also are similarities. Both forensic psychiatrists and forensic psychologists can serve as expert witnesses in the courtroom. Their specialty permits them to address medicolegal issues such as the competency of an accused person to stand trial (CST) where it must be determined that the accused understands the charges and legal process brought against him or her and that he or she is able to assist the representing defense lawyer (Fortunati et al., 2006). Both disciplines have expertise in evaluating the mental state of the perpetrator at the time of the offense (MSO), child custody and child abuse cases, and other legal specifics. Psychiatrists and psychologists have made major contributions to the development of crisis/hostage negotiation techniques and have performed a variety of roles and functions within these areas. These techniques, when implemented by police agencies, have sharply reduced both injuries and loss of life in field situations (Hatcher et al., 1998).

Forensic consulting in neuropsychology begins like many other aspects of clinical evaluation practice, by collecting and comparing raw data with normative data from neuropsychological tests. Examples of such tests are the Wechsler Adult Memory Scale (WMS), the Wechsler Adult Intelligence Scale (WAIS), and the Wechsler Intelligence Scale for Children (WISC). Other tests include the Halstead–Reitan Neuropsychological Battery, the Boston Naming Test, the Wisconsin Card Sorting Test, the Benton Visual Retention Test, and the Controlled Oral Word Association. This scientific approach to the investigation of brain–behavior relations provides forensic neuropsychology its unique professional standing (Kaufmann, 2009). Both forensic psychiatrists and forensic psychologists work in four major branches of the law: civil law, criminal law, family/domestic law, and regulatory law. In the aspect of dealing with the criminal justice system, the types of forensic evaluation done by forensic psychiatrists (Rappeport, 1982) and forensic psychologists are similar. The testimony of mental health experts is important evidence considered by the criminal courts to determine questions arising throughout the adjudication process; most commonly competence to stand trial (CST), criminal responsibility or legal insanity, and sentencing in capital and non-capital cases (Table 2; Redding et al., 2001).

**Table 2:** **Questions that arise during the adjudication process**.

Criminal proceedings	Civil proceedings
Malingering	Malingering
Competency to stand trial	Personal injury
Waiver of Miranda rights	Mental disability
Criminal responsibility	Professional malpractice
Death penalty mitigation	Civil commitment
Impact of mental illness or substance abuse on behavior	Employment discrimination

A brief description of some forensic services will help illuminate the role of the forensic expert:

### Competency to stand trial

These are cases where a crime has been committed and the suspect usually is already incarcerated and awaiting trial. The legal question for the courts is whether or not the suspect is competent to stand trial in a court of law. The forensic psychiatrist or the psychologist is retained by an attorney or the court for the purpose of determining whether the defendant meets minimal criteria for competency to stand trial according to the Code of Criminal Procedure of the State in which the defendant would stand trial.

In the landmark case, *Dusky vs. United States, U.S. Supreme Court, 1960*^5^^,^^6^, the U.S. Supreme court established the standards for competence to stand trial as whether the accused has: Rational as well as factual understanding of the proceedings against him/her, sufficient present ability to consult with his/her attorneys with a reasonable degree of rational understanding, and thus: whether the defendant understands the nature of the legal process, the charges currently pending in his/her case, the possible consequences of said charges, and the defendant’s capacity to assist counsel and participate in his/her own defense.

The forensic psychiatrist/psychologist take a detailed medical, psychiatric, and social history. All relevant legal and medical documents are reviewed. Collateral sources are contacted for information relevant to the case, or to the defendant. A mental status examination (MSE) is performed. Often, the forensic psychologist will perform additional psychological assessments, such as the Minnesota Multiphasic Personality Inventory (MMPI-II) to aid the forensic expert in reaching an inclusive opinion of the defendant’s overall functioning. The forensic psychiatrist/psychologist then writes a formulation based upon response to the legal question. Defense attorneys use this document to make their case in court. Frequently, the forensic psychiatrist/psychologist is required to testify in court.

Below is a description of a forensic psychiatrist’s evaluation and court testimony in the case of Robert Alan Fratta, who in 1994 was accused of murder for hire. Robert Fratta hired Joseph Prystash, who hired the killer, Howard Guidry. Farah Baquer Fratta, mother of three young children, was shot twice in the head after driving into her garage. Farah and her husband, Robert Fratta, were in the middle of an adversarial divorce. Fratta tried to collect life insurance worth over $200,000 after Farah’s death. Police arrested Fratta and the two men he had hired to kill his wife. Eventually all three defendants were found guilty and sentenced to death. A retrial was ordered due to “constitutionally inadequate testimony.” A forensic expert was requested to evaluate Robert Fratta’s criminal responsibility and the expert^7^ concluded that Robert Fratta met the statutory requirement for mitigation in his current capital murder case. Despite these findings, the defendant’s conviction was upheld. The convicted Robert Fratta remains on death row awaiting execution or acquittal based on his attempts at retrial^8^.

### Forensic psychiatric evaluation of a sexually violent predator

There is a high prevalence of psychiatric disorders in the sexual offender populations, especially in forensic psychiatric settings (Koch et al., 2011). Persons who commit sexual crimes have a high rate of psychiatric disorders (Dunsieth Jr. et al., 2004; Fazel et al., 2007). A Sexually Violent Predator or a sexually dangerous person (SVP/SDP) is a designate for a group of extremely dangerous incarcerated sex offenders who represent a threat to public safety if released from custody. A sexually violent offense is defined by legal statute as sex crime felony convictions, such as child molestation, sodomy, or rape (Sreenivasan et al., 2003, 2010; Carlsmith et al., 2007). The purpose of this type of evaluation is usually to determine whether the defendant suffers from a behavioral abnormality that makes him engage in a predatory act of sexual violence, the defendant meets the criteria for the particular State’s Violent Predator Statute, the defendant is inclined to perpetrate the act again and recommend appropriate psychosocial behavioral interventions or strategies which could potentially be effective in preventing relapse and recidivism.

In the landmark case, *Allen vs. Illinois, U.S. Supreme Court, 1986*, the Supreme Court held that in order to declare an accused person a SDP, the state must in addition to proving the commission of a sexual assault, prove the existence of a mental disorder for more than one year and a propensity to recommit sexual assaults. The Supreme Court further held that treatment, not punishment should be provided for persons adjudged sexually dangerous (see text footnote 5 and 6).

### Child custody

The Judge in the landmark case *Painter vs. Bannister*, *Supreme Court of Iowa, 1996*, used “The Best Interest of the Child” as the standard to determine the custody of a child. All parties in the child custody case were found fit, but the court accepted the validity of the child psychologist’s testimony to rule in favor of the grandparents. Emphasis was placed on the grandparents’ ability to provide a “stable, dependable, conventional, middle class, middle west background,” as opposed to the father who was viewed as a “Bohemian^9^” In this landmark case, the forensic psychologist used evidence from interviews and records of the child, information about the Bannisters, including appropriate testing of and “in depth interviews” with Mark – the child in question.

## The Collaboration between Forensic Psychiatrists and Forensic Psychologists

The potential for an incorrect assessment is always a possibility among forensic psychiatrists and forensic psychologists; as such, collaboration between these two professionals can add credence to the professions (Grisso, 1987). Their collaboration will alleviate the erosion of their credibility (Grisso, 1993). The distinct differences within educational background, practice experiences, and methodological emphasis can complement the services provided by both professionals. This collaboration also may begin to address some of the perceived dissension, competition, and opposition to licensure issues that currently exist between the professions. Additional benefits of psychiatrists and psychologists collaborating and conferring on cases would build mutual respect, joint validation, and improved communication among the professions. By working and conferring together, their intersection of training and practice solidifies their foundational base and eliminates individual challenges and deficiencies that currently exist. Clinical care is best delivered with the integration of many perspectives, including psychiatry, psychology, social work, occupational therapy, spiritual understanding, education, and recreation (Simpson and Chaplow, 2001). This combination of professional knowledge and experiences truly serves as strength rather than a deficiency.

## Employment

The employment opportunities for psychologists are multitudinous. They are found in the private and public sector and in all work domains, as demonstrated by Table 3.

**Table 3:** **Employment by industry, occupation, and percent distribution 2008 and change projected 2018**.

Occupational Title	Employment, 2008	Projected employment, 2018	Change, 2008-2018	
			Number	Percent
**Occupational opportunities for psychologists for 2008 and 10 year projection**				
Psychologists	170,200	190,000	19,700	12
Clinical, counseling, and school psychologists	152,000	168,800	16,800	11
Industrial-organizational psychologists	2,300	2,900	600	26
Psychologists, all other	15,900	18,300	2,300	14

ftp://ftp.bls.gov/pub/special.requests/ep/ind-occ.matrix/occ_pdf/occ_19-3030.pdf

National Employment Matrix, Bureau of Labor Statistics

In 2008, psychologists held 170,200 jobs. 29% of these psychologists worked in academia: teaching, counseling, testing, research, or administration. Twenty-one percentage were employed in healthcare, primarily in offices of mental health practitioners, hospitals, physicians’ offices, and outpatient mental health and substance abuse centers. Government agencies at the State and local levels employed psychologists in correctional facilities, law enforcement, and other settings. Like psychologists, psychiatrists have a strong presence in industry (see Table 4 below).

**Table 4:** **Occupational employment and wages May 2010**.

Industry	Employment (1)	Percent of industry employment
**Industry with the highest level of employment for of psychiatrists 29-1066 (psychiatrists) May 2010**		
Offices of physicians	5,460	0.24
Psychiatric and substance abuse hospitals	4,420	1.83
Outpatient care centers	3,230	0.55
General medical and surgical hospitals	3,070	0.06
Local government (OES designation)	1,540	0.03

*Source: occupational employment statistics*.

**Figure 1:**
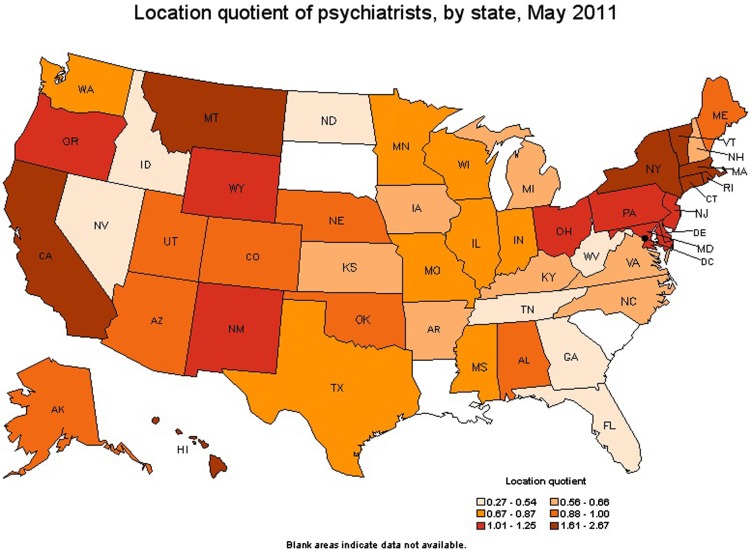
**Geographic profile for psychiatrists by state (http://www.bls.gov/oco/ocos056.htm, 2010-2011)**.

The American Medical Association (AMA) data showed that in 2007, about 75% of physicians in patient care were located in metropolitan areas while the remaining 25% were located in rural areas (see text footnote 2). See Figure 1: Psychiatrists by State.

## Summary

There is natural overlap between the work of forensic psychiatrists and forensic psychologists. Forensic Psychiatrists assess the level of criminal and legal responsibility of defendants in cases of fraud, embezzlement, murder, physical aggression, and other crimes and court proceedings. In order to evaluate inmates for release, forensic psychiatrists, and forensic psychologists study the risk factors for repeat criminal behavior and research the neurobiological aspects of psychopathic personalities to predict possible threats to society at large. Forensic psychologists assess litigants in custody disputes and insurance claims. In family courts they offer psychotherapy services, perform child custody evaluations, investigate reports of child abuse, and conduct visitation risk assessments. Forensic psychologists in civil courts frequently assess mental competency, serve as alternate expert witnesses, and provide psychotherapy to crime victims; those in criminal courts conduct evaluations of competency to stand trial, work with child witnesses to crimes, and provide assessment of juvenile and adult offenders for sentencing.

The forensic psychologist thinks “assessments” and uses these assessments to reach a diagnosis and conclusion. The forensic psychiatrist on the other hand, gathers information and uses assessment tools combined with his knowledge of psychiatry to formulate a diagnosis that answers the legal question, or the purpose of the evaluation. Here lies the fundamental basis for the similarity of the two professions. A collaborative effort on the part of both professionals will serve both the client and profession well. This collaboration also may begin to address some of the perceived dissension, competition, and opposition to licensure issues that currently exist between the professions.

## Conflict of Interest Statement

The authors declare that the research was conducted in the absence of any commercial or financial relationships that could be construed as a potential conflict of interest.

## Footnotes

^1^www.aapl.org/ethics. (2005, May). Retrieved October 13, 2011, from American Academy of Psychiatry and the Law

^2^http://www.bls.gov/oco/ocos056.htm. (2010-2011). Retrieved 10 2011, from Occupational Outlook Handbook, 2010-11 Edition.

^3^http://www.guidetopsychology.com/psypsy.htm. (n.d.). Retrieved October 2011.

^4^www.diffen.com/difference/Psychiatrist_vs_Psychologist. (n.d.). Retrieved 2011

^5^Dusky Vs United States 363 U.S 402, 504 (United supreme court April 18, 1960)

^6^Alllen Vs Illinois 478 U.S.364, 85-5404 (U. S. Supreme Court 1986)

^7^Expert witness Report from Robert Fratta’s evaluation. (n.d.).

^8^www.hcdistrictclerk.com. (n.d.). Retrieved from 11950440101A – the State of Texas vs. Fratta, Robert Alan (Court 230)

^9^Bannister Vs Painter 258 Iowa 1390 (1996)
